# Injuries of the Knee-Joint

**Published:** 1857-04

**Authors:** Benjamin Woodward

**Affiliations:** Sharon, Ill.


ARTICLE III.—*Injuries of the Knee-Joint.* By BENJAMIN WOODWARD, M.D. of Sharon, Ill.

On the evening of the 4th of April last, I was sent for, in conjunction with Drs. Morey and Pomeroy, of Geneseo, to see George Rose, a German, twenty-one years of age, who had received a severe injury of the right knee, from a large circular saw. We found the whole soft parts of the knee torn into shreds. The outer condyle of the femur was cut through. The patella was also fractured, and nearly one-half of it torn loose from its attachments, and had to be removed. The whole cavity of the joint was filled with saw-dust, and the integuments so much torn that, after removing no more than was absolutely necessary, we could by no means close the wounds. As the patient was young, vigorous, and of good habits, we concluded not to amputate, extensive as the wound was, but to endeavor to save the limb. We, therefore, cleaned and dressed the wound, and secured the limb as for compound fracture. The case was from this time left in my hands. Absolute immobility of the limb was secured, and water-dressings and a low diet depended on.

On the second day violent inflammatory action took place; this was combated by blood-letting, and mercurials and opium. To this treatment the inflammation yielded, and about the sixth day suppuration commenced. After a time this became so excessive that it became necessary to resort to the free use of quinine and the mineral acids. I also had the wound syringed twice a-day with a solution of tannin in water; he was also put on a generous, but unirritating diet.

Varying the treatment from time to time, as circumstances indicated, he so far recovered, that by the 1st of October he was able to walk with the aid of a crutch; and, at the present time, is able to be at work, but with complete anchylosis of the joint.

Throughout the whole of the treatment, the greatest difficulty was with the condyle of the femur, the suppuration seeming to prevent union of the bone, and union did not take place till the suppuration became much less profuse.

A somewhat similar case occurred to me in February, 1850. A Mr. Rowe, a young man of good health and habits, was wounded by the bursting of the breech of an old musket, which was heavily loaded, and rammed with pounded brick. This was laid on the ground, and R. stooped down to fire it, when it exploded, and the whole charge struck his knee. The upper part of the tibia was injured, and a piece of iron one and a half inches long and half an inch wide, and the thickness of the gun-barrel, was forced under the patella. The whole knee was much injured.

This case I treated much as the first detailed, and the result was recovery, with partial stiffness of the joint.

Injuries of the knee-joint are of so serious a character, that it is generally thought that amputation is the safer course; but I think I am warranted in the belief, that where the general health of the patient is good, and his habits have been regular, and especially if he have the advantage of youth on his side, many such cases may be cured without resort to the knife. I have in many cases found the free application of water, the best means for combating local inflammation, and I have as often found the solution of tannin efficacious in moderating profuse suppuration.

